# Sexual harassment and implicit gender-career biases negatively impact women’s life expectancy in the US: a state-level analysis, 2011–2019

**DOI:** 10.1186/s12889-024-18450-9

**Published:** 2024-04-23

**Authors:** George B. Cunningham, Pamela Wicker

**Affiliations:** 1 Laboratory for Diversity in Sport, Department of Sport Management, University of Florida, Gainesville, USA; 2 Department of Sport Science, Bielefeld University, Bielefeld, Germany

**Keywords:** Sexual harassment claim, Implicit bias, Health, Female, Gender

## Abstract

**Background:**

Despite some gains, women continue to have less access to work and poorer experiences in the workplace, relative to men. The purpose of this study was to examine the relationships among women’s life expectancy and two work-related factors, sexual harassment and gender-career biases.

**Method:**

We examined the associations at the state level of analysis (and District of Columbia) in the US from 2011 to 2019 (*n* = 459) using archival data from various sources. Measures of the ratio of population to primary health providers, year, the percent of adults who are uninsured, the percent of residents aged 65 or older, and percent of residents who are Non-Hispanic White all served as controls.

**Results:**

Results of linear regression models showed that, after accounting for the controls, sexual harassment and gender-career biases among people in the state held significant, negative associations with women’s life expectancy.

**Conclusion:**

The study contributes to the small but growing literature showing that negative workplace experiences and bias against women in the workplace negatively impact women’s health.

## Introduction

Despite some gains, women continue to have less access to work and poorer experiences in the workplace, relative to men [1]. For example, even though women in the US are better educated than men, they are paid less and are less likely to hold top leadership positions [2–4]. Furthermore, women who do obtain key leadership roles are frequently placed in precarious positions, tasked with leading units in crisis or with a history of poor performance [5, 6]. Women are also likely to encounter stereotypes about their performance [7], confront prejudice [8], and face various forms of mistreatment [9, 10].

Given these patterns, it is not surprising that gender differences emerge when considering work-related outcomes. When people routinely encounter obstacles to and mistreatment in their work, they might alter their aspirations, perform in ways that correspond with expectations and stereotypes of them, or disengage from their work [11]. Consistent with this view, research evidence suggests women have fewer aspirations to pursue leadership roles after they have experienced injustices in the job search process [12]. Gender differences are also present in voluntary turnover, with the quality of work and support largely explaining these patterns [13, 14].

Poor work experiences also have spillover effects, such that they can influence nonwork outcomes, including people’s health and wellbeing. For example, Hackett and colleagues collected data from women in the UK, asking about their experiences with discrimination and mental health [10]. Women who encountered discrimination also reported lower wellbeing (i.e., more distress, poor mental functioning, and poor life satisfaction) at the time, but the effects carried over four years later, too. These findings align with related research showing that experiences with mistreatment and discrimination can negatively affect one’s psychological and social wellbeing [15–17].

In the current study, we expand this work in several ways. The existing scholarship has examined individuals’ workplace experiences and their subsequent wellbeing. However, patterns of opportunity or mistreatment can take on a shared property and can subsequently shape the outcomes for members of that community [18]. For example, Payne et al. showed how racial biases in a county related to the economic wellbeing among Black community members [19]. Similarly, Sitzmann and Campbell examined religiosity of communities in the US and around the world and found that as collective religiosity increased, so too did gender differences in work opportunities and wages [20].

Drawing from this work, we examine the associations between work-related factors and women’s life expectancy. Specifically, we consider two measures of work at the state level of analysis: the prevalence of sexual harassment and bias against women working. Concerning the outcome of interest, previous researchers have focused on varied measures of wellbeing, such as stress, anxiety, and depressive symptoms. Each of these mental health factors is associated with decreased life expectancy [21, 22]. Our approach of focusing on broader communities afforded us to the opportunity to examine this possibility. Specifically, we considered the relationships among state-level measures of work and the life expectancy among women in the state. We found that sexual harassment and bias against women in the workplace are linked with lower life expectancy among women. In the following sections, we present our theoretical framework and specific hypotheses.

The research also has implications for practice. Consistent with the social determinants of health (SDOH) model [23, 24], public health officials looking to improve women’s life expectancy should consider the influence of the economic and social opportunities and resources. Making work more accessible and inclusive for women is not only good practice [25, 26], but will also likely yield health benefits. Specifically, our findings suggest that sexual harassment and implicit bias against women at work might not only decrease working conditions, but these factors also result in poorer health and lower life expectancy, respectively. In turn, reduced health can be associated with economic costs for both public health systems and employers. Given these indirect effects, taking measures for addressing sexual harassment issues at work and biases against women and ultimately improving the situation for women is important. Importantly, since our data are not at the individual level, but at an aggregate (state) level, the findings indicate that these patterns are not only present for a few individuals. In fact, there are deeper structures and working cultures across states that create such a working environment, which might take a while to be changed.

## Theoretical framework

### Work and community predictors of health

The social determinants of health (SDOH) model suggests that people’s health is impacted by personal factors, such as their genetics and health-related behaviors, but also factors external to the individual [23, 24]. The medical care to which they have access and the quality of that care, the living conditions of their homes and broader communities, and economic factors, among others, all have the potential to impact people’s health and wellbeing collectively and synergistically. As some examples, the representation of Black primary care physicians in a county is associated with life expectancy among Black people in the US [27]. In Taiwan, increased green space links with decreased risk of bipolar disorder and disability-adjusted life years [28]. Further, air quality and water pollution negatively affect physical health, though these patterns can be offset when people have access to physical activity [29].

Work is another social determinant that can affect people’s health [30]. The Allostatic Load model helps explain the underlying mechanisms [30, 31]. When encountering stressful situations at work, the body will have psychological (e.g., fear), physiological (e.g., cortisol), and psychosomatic (e.g., fatigue) responses. As the stress continues, people develop new setpoints, or what is known as the secondary Allostatic Load process. The new setpoint can affect people’s immune system (e.g., immunoglobulin levels), cardiovascular (e.g., resting blood pressure), and metabolic (e.g., cholesterol) responses. Over time, chronic stress can then negatively impact health outcomes, such as depression, diabetes, and all-cause mortality. These are considered tertiary Allostatic Load responses. Ganster and Rosen’s review of the literature pointed to strong support for primary outcomes, with some support for the effects of workplace stressors on secondary and tertiary outcomes, too [30]. More recently, researchers have demonstrated the links among stress-inducing work experiences, such as incivility, discrimination, and mistreatment, and subsequent health outcomes, including poor physical and psychological wellbeing and unhealthy behaviors [32–35].

### Community-level effects

Much of the work related to work- and community-related factors impacting people’s health has focused on the individual-level outcomes. However, when social determinants of health are pervasive and consistent within a given setting, it is possible that the factors would affect people in that setting similarly. In this case, the social determinants and the associated health outcomes take on a shared property. These possibilities are consistent with Hood et al.’s work focusing on county-level health in the US [36]. They theorized and offered empirical support for the notion that economic and social factors could meaningfully contribute to the collective health among the county residents.

Consider the following examples that illustrate the link between work-related stressors and collective health outcomes. Incivility is commonly considered at the individual level and negatively affects people’s work and life outcomes [37]. Smittick and colleagues, however, showed that these patterns can also take on a shared property among members of work groups, negatively affecting collective psychological outcomes [38]. Beyond the organization focus, researchers have shown that county-level work-related indicators are predictive of county-level health outcomes. Goetz and colleagues, for example, showed that measures of social capital, education, and self-employment were linked with fewer poor mental health days among county residents in the US [39]. Likewise, at the county-level of analysis in the US, a gender-wage gap is positively associated with domestic violence [40]. This research suggests that, while work-related social determinants of health impact individuals, they can also take a shared property and impact the collective health of broader communities.

### Current study

Building from this work, we examine the relationships among two work-related factors (sexual harassment and bias toward women at work) and women’s life expectancy at the state level. Consistent with the Allostatic Load model, these factors can negatively affect women’s physical, psychological, and social well-being and, thus, have the potential to reduce their life expectancy. We outline each of the relationships in the following space.

Sexual harassment represents “behavior that derogates, demeans, or humiliates an individual based on that individual’s sex” (Berdahl, 2007, p. 641). Researchers have commonly focused on three areas: unwanted sexual attention, sexual coercion, and gender harassment [42, 43]. Though the former two are most evident to observers, gender harassment is more prevalent in practice, and paradoxically, organizational policies aimed at reducing sexual harassment are weakest in this area [44]. Cortina and Areguin [44] outlined several harmful effects of sexual harassment, including a decrease in job attitudes, increased work withdrawal, and poor job performance. Of particular relevance to the current study, the authors also showed how sexual harassment is related to poorer health, including lower levels of wellbeing, symptoms and posttraumatic stress, poor health behaviors, and increased stress.

Importantly, sexual harassment can also take on a shared property at the community and state levels, though research in this area is scarce [45]. For example, King and colleagues examined sexual harassment in one state in the US (Idaho). They observed that 20% of people working in the state experienced some form of sexual or gender harassment, and the patterns remained stable over time [46]. Further, Cortina and Wasti [47] conducted a cross-cultural study and found that strategies for coping with sexual harassment varied among White Americans and people from Turkey. Their research suggests that ways people think about and respond to sexual harassment could be commonly understood among people in a given environment. Finally, O’Neal and colleagues [45] commented on the potential public health benefits of addressing sexual harassment within a cultural context. They noted, “challenging complex issues such as male entitlement, rigid gender norms, and the subjugation and objectification of women that arise from patriarchal power structures is likely to benefit women’s health“ (p. 2588).

The theoretical and limited empirical work related to sexual harassment is consistent with related scholarship at the state level of analysis showing that harassment and mistreatment can negatively impact people’s health. For example, gender inequality in a state, as reflective in reproductive health rights, work participation, and empowerment among women, is related to both psychological and physical intimate partner violence in the state [48]. Hatzenbuehler and colleagues found that immigrants living in states with restrictive policies, which potentially resulted in harassment and mistreatment, experienced poor health outcomes [49].

Collectively, this scholarship suggests that sexual harassment is related negatively to physical, psychological, and social health outcomes, and that the patterns of sexual harassment can vary based on context. Given that poor psychological health and health behaviors are linked with premature death [21, 22], we hypothesized the following:

**Hypothesis 1**

State-level rates of sexual harassment will be negatively associated with women’s life expectancy in the state.

Next, we consider state-level implicit biases related to women at work. Unlike explicit forms of bias, which are deliberate and consciously maintained [50], implicit bias represents the automatic, unintentional associations people make with different targets [51]. They are likely to manifest when there is congruence between a target (e.g., women in the workforce) and subsequent evaluations people have toward that target (e.g., good or bad) [52]. People’s implicit biases activate automatically, though there is some evidence that people can predict their own biases with some accuracy [53, 54].

Though scholars have historically considered implicit bias at the individual level, recent evidence points to the value of considering aggregate-level bias and its association with subsequent outcomes [18]. From this perspective, although individuals will hold their own biases, people within a given social environment are also exposed to similar sets of cues, activities, and experiences. As such, biases have the potential to take on a shared property, and the collective biases in one community might vary from those in another. Further, relative to the experiences of an individual, environmental factors are stable, and thus, are likely to be better predictors of subsequent outcomes. Consistent with this view, researchers have shown that community-, state-, and country-level bias is predictive of a host of outcomes, including COVID-19 cases and deaths [55], racial disparities in the use of police force [56], patterns of school discipline [57], girls’ science and math achievement [58], and organization’s inclusion strategies [59].

In the current study, we focused on implicit gender-career biases. These biases reflect a stronger connection between women and family than between women and careers outside the home. People across a host of contexts hold such biases, including college students in Korea [60], surgeons around the world [61], and parents in the Netherlands [62], among others. This previous research has shown how gender-career implicit attitudes relate to women’s guilt associated with working outside the home [62] and career decisions [60]. Furthermore, a study of Indian journalists revealed that awareness of implicit biases reduced the incidence of gender-biased content [63]. Relatedly, Teelken and colleagues showed how implicit gender biases helped perpetuate the social mobility and career outcomes that limit women professors [64].

Collectively, this scholarship suggests that implicit gender-career biases relate to how women and men engage with their work and work outcomes for women. Further, implicit biases can take on a shared property at the community, state, or national levels. These associations give rise to the possibility that people in a given state might hold shared implicit gender-career biases, and that these biases might negatively impact women’s health and wellbeing. Thus, we hypothesized:

**Hypothesis 2**

State-level implicit gender-career biases will be negatively associated with women’s life expectancy in the state.

## Methods

### Data collection and variables

To test the study hypotheses, US state-level data were collected from various sources. Since data were only available from 2011 to 2019, this period represents the data period of the present study. Table 1 gives an overview of the variables included in this study and their summary statistics. The data that support the findings of this study are available from the corresponding author upon reasonable request.

**Table 1:** Overview of variables and descriptive statistics

Variable	Description	Min	Max	Mean	SD
Life Expect	Women’s expected number of years to live	77.57	85.10	81.08	1.63
Harassment	Number of claims in a state filed by women, per million women living in the state	1.46	148.96	53.52	26.82
Bias	Implicit bias score related to women at work (relative to at home)	0.28	0.48	0.38	0.03
Year	Year of the data	2011.00	2019.00	2015.00	2.59
Primary Care Ratio	Ratio of population to primary health providers	479.73	1919.89	1291.75	243.28
Uninsured Adults	Share of adult who lack health insurance	0.04	0.32	0.17	0.06
Percent 65older	Share of the population aged 65 or older	0.07	0.20	0.14	0.02
Percent White	Share of the population who identify as Non-Hispanic White	0.22	0.96	0.70	0.16

The outcome of interest is women’s life expectancy (*Life Expect*) which was gathered from VizHub [65].

Information about the number of female sexual harassment (*Harassment*) claims by state was obtained from the US Equal Employment Opportunity Commission [66]. We computed the number of claims in a state filed by women, per million women living in the state.

Implicit bias (*Bias*) scores were obtained from the Project Implicit dataset that are anonymized and made publicly available [67]. Users can visit the Project Implicit site to take assessments of their biases toward different groups, including the links between women and work. The site administrators then de-identify the data and make them publicly available. Other researchers examining biases at the aggregate level, whether community, county, state, or nation, have also drawn from this dataset [57, 58, 68, 69]. In the current study, we focused on their gender-career implicit biases, as measured by the Implicit Association Test, or IAT [70]. As Greenwald et al. [71] explained, the IAT assesses a person’s “response latencies” to determine the strength between two concepts (p. 18). Test scores can range from − 2 to + 2, though almost all participants score between − 1 and + 1. Higher scores are reflective of more gender-career implicit biases.

Finally, we included several control variables that might otherwise affect women’s life expectancy, all of which were collected from the County Health Rankings & Roadmaps site [72]. We included the year of data collection (*Year*) to account for the effects of time, treating it as a continuous variable. We also included ratio of population to primary health providers (*Primary Care Ratio*). We included this control owing to research showing that limited access to medical professionals can result in poor health outcomes [73, 74]. Next, given that a lack of health insurance links with shorter life expectancy [75, 76], we also controlled for the percent of adults who are uninsured (*Uninsured*). Finally, the characteristics of a state’s residents [77–79], including their age and race, can impact health outcomes, so we controlled for percent of residents aged 65 or older (*Percent 65older*) and the percent of residents who are Non-Hispanic White (*Percent White*).

Altogether, 9 years of data (2011–2019) for 50 states and the District of Columbia were gathered, leading to a total number of *N* = 459 observations on a state-year basis.

### Empirical analysis

The empirical analysis strategy is based on a set of regression analyses where women’s life expectancy serves as the dependent variable. Given that this variable is a continuous measure, linear regressions (ordinary least squares) were run. The remaining variables from Table 1 were included as independent variables.

We first computed means and standard deviations, as shown in Table 1. To assess potential multicollinearity of independent variables, bivariate correlation analyses were run (Table 2). All correlation coefficients were below 0.8, the proposed threshold by Hair et al. [80].

**Table 2:** Correlation matrix of independent variables

Variable	1	2	3	4	5	6	7	8
1. Life Expect	---							
2. Harassment	− 0.29***	--						
3. Bias	− 0.16***	0.17***	--					
4. Year	0.02	− 0.13**	− 0.75***	--				
5. Primary Care Ratio	− 0.42***	0.20***	− 0.04	0.22***	--			
6. Uninsured Adults	− 0.37***	0.24***	0.35***	− 0.41***	0.49***	--		
7. Percent 65older	0.05	− 0.20***	− 0.32***	0.49***	− 0.07	− 0.38***	--	
8. Percent White	− 0.10*	− 0.33***	0.12*	− 0.11*	− 0.10*	− 0.20***	0.24***	--

Note: *p <.05. **p <.001. ***p <.001

We tested the hypotheses through weighted OLS regression using SPSS Version 29. Women’s life expectancy was included as the dependent variable. Given the variability in the number of responses per state, which is also driven by differences in state population, some scholars have advocated for weighting the analyses to account for this issue [58]. To account for the differences in the number of responses per state, the procedure suggested by Nosek et al. (2009) was employed. Specifically, the log of the inverse weights based on standard errors was computed. The resulting variable was used as a weighting variable in the empirical analysis to provide more reliable estimates in the states with a higher number of responses to the implicit bias test. This approach has already been employed in previous research, too [55]. Further, and also consistent with Nosek et al., we standardized the controls and independent variables. Multicollinearity was also checked using variance inflation factors (VIFs). Since all VIFs were far below the suggested threshold of 10 [80], multicollinearity should not present an issue in the present analysis.

It could be argued that the above empirical analysis might be affected by simultaneity and causality issues as all variables are measured in the same year. To address this issue, the above set of models was re-esteemed with lagged effects for the sexual harassment and implicit bias variables (as unemployment is already measured at the beginning of each year). The direction and significance of the coefficients of the variables of interest (sexual harassment and working conditions) remain the same, suggesting that the present findings have some robustness in this regard.

## Results

The descriptive statistics in Table 1 indicate that women’s life expectancy in the US is on average 81.08 years (*SD* = 1.63), with a range from 77.57 to 85.10 years. On average, there were 53.32 claims per million women in a state (*SD* = 26.82). The average implicit bias score is 0.38 (*SD* = 0.03) across states, with state values ranging from 0.28 to 0.48.

Table 3 displays the results of the weighted OLS regression. As seen in Model 1, the controls accounted for 24% of the variance in *Life Expectancy*. After accounting for these effects, *Harassment* and *Bias* contributed 9% unique variance (*p* <.001).

**Table 3:** Linear regression predicting women’s life expectancy

	Model 1			Model 2		
Variable	*B*	*SE*	***p***-value	*B*	*SE*	***p***-value
Year	− 0.10	0.17	0.54	− 0.69	0.22	0.001
Primary Care Ratio	− 0.50	0.10	< 0.001	− 0.32	0.09	< 0.001
Uninsured Adults	− 0.42	0.10	< 0.001	− 0.48	0.10	< 0.001
Percent 65older	− 0.01	0.08	0.94	0.03	0.08	0.682
Percent White	− 0.36	0.08	< 0.001	− 0.50	0.08	< 0.001
Harassment				− 0.46	0.07	< 0.001
Bias				− 0.26	0.10	0.008
Model	*R*^2^ = 0.24, *p* <.001			Δ *R*^2^ = 0.09, *p* <.001		

Hypothesis 1 predicted that state-level rates of sexual harassment would be negatively associated with women’s life expectancy in the state. As seen in Table 3, Model 2, the association between *Harassment* and *Life Expectancy* was significant and negative. The more claims from women (per million women in the state), the lower the life expectancy for women in the state. Thus, Hypothesis 1 was supported.

With Hypothesis 2, we predicted that state-level implicit gender-career biases would be negatively associated with women’s life expectancy in the state. This hypothesis was supported (see Table 3, Model 2), as the association between *Bias* and *Life Expectancy* was significant and negative.

Finally, several of the control variables demonstrated meaningful effects. Women’s *Life Expectancy* decreased when there were fewer primary care physicians (*Primary Care Ratio*), as the share of *Uninsured Adults* increased, and as the share of Non-Hispanic White (*Percent White*) residents decreased.

## Discussion

Women commonly encounter barriers to meaningful employment, and previous researchers have shown the effects on women’s work-related outcomes. Drawing from various theoretical lenses, including the SDOH [23, 24] and Allostatic Load model [30, 31], the purpose of this study was to expand the current scholarship by considering state-level work factors and their impact on women’s life expectancy. Consistent with our theorizing, we found that women’s life expectancy was lower when the state had higher levels of sexual harassment and when people in the state endorsed women-career implicit biases. In the following space, we discuss the contributions, implications, limitations, and future directions.

### Contributions and implications

The study makes several contributions to the literature. First, in states with work environments that are hostile toward women– as measured by harassment and collective implicit biases– the women in that state will suffer poor health outcomes. Previous researchers have shown that sexual harassment can result in poor work and health outcomes [44]. We extend this work by also showing that collective implicit biases about women at work can harm women’s health, too.

Second, whereas previous researchers have shown that people in various contexts endorse implicit gender-career biases [60–62] and that collective gender-related biases relate to academic achievement [58], our study extends this scholarship by demonstrating that collective gender-career biases can harm women’s life expectancy. These findings are consistent with related scholarship showing the pernicious effects of community-level racial biases on residents’ health [55, 81, 82].

Third, our study demonstrates the importance of considering aggregate-level factors that potentially relate to women’s life outcomes. Certainly individual-level interactions can and do impact women. But looking at collective patterns– in this case, at the state level– can also help illuminate configurations of disrespect, mistreatment, and bias. Indeed, consistent with Payne et al.’s notion of “bias of crowds” (Payne et al., 2017, p. 237), our work shows how employment-related behaviors, implicit biases, and opportunities can take on a shared property and collectively impact women’s health.

### Limitations and future directions

The current study has some limitations that can represent avenues for future research. Our research is limited to the available data at the state level. For example, the study can only consider sexual harassment claims that were officially reported and hence recorded. However, it is not clear if the number of reported claims is similar to the sexual harassment that actually occurred or if it is tip of the iceberg. Relatedly, previous researchers have shown that sexual harassment is under-reported [83–85]; thus, the figures we show in Table 1 are likely an underrepresentation of how women in a given state encounter harassment. Moreover, as per the theoretical discussion, women’s life expectancy is affected by further factors, which are however, not included in our models. Again, data availability across the US and at the state level represents a limitation in this respect. Future research would benefit from obtaining data on further factors that might affect women’s life expectancy. Likewise, exploring the effects of our two work-related on other outcomes, such as different physical and psychological health outcomes, would be a fruitful perspective.

## Conclusion

The current study investigated the association between two work-related factors (i.e., sexual harassment at work and implicit bias against women at work), and women’s life expectancy using 9 years of state-level data in the US. The study shows the substantial negative impact of poor work experiences for women. The findings point to the urgency with which policy makers, organizational leaders, and public health officials need to improve the work environment for women– changes that have the potential to impact their life expectancy.

## Data Availability

All data came from publicly available sources: life expectancy (https://vizhub.healthdata.org/gbd-results/); sexual harassment claims (https://www.eeoc.gov/data/eeoc-sexual-harassment-charges-state-gender-fy-1997-fy-2021); gender-career implicit bias (https://osf.io/y9hiq/); and unemployment (https://fred.stlouisfed.org/release?rid=112_t=sa%3Bstate_ob=pv_od=desc). The control variables were collected from the County Health Rankings and Roadmap (https://www.countyhealthrankings.org).
